# Value added by an inter-continental cancer consortium

**DOI:** 10.18632/genesandcancer.215

**Published:** 2021-05-24

**Authors:** Mahadev Rao, Prasanna Venkatraman, Debabrata Mukhopadhyay, Susanta Roychoudhury, Nathan L. Vanderford, Vivek M. Rangnekar

**Affiliations:** ^1^Manipal Academy of Higher Education, Manipal 576104, India; ^2^Tata Memorial Centre, ACTREC, Mumbai 400012, India; ^3^Mayo Clinic College of Medicine and Science, Jacksonville, Florida 32224, USA; ^4^Saroj Gupta Cancer Center and Research Institute, Kolkata 700063, India; ^5^Markey Cancer Center, University of Kentucky, Lexington, Kentucky 40536, USA

## Summary

The United States of America reports almost double the number of cancer cases relative to India that has a much larger overall population. Accurate insights into such differences in cancer rates may be provided by a better understanding of the genetic and lifestyle influences on tumor growth, progression, heterogeneity, and the underlying stressors that prompt pre-clinical dormant lesions to progress into malignant tumors, as well as by synchronizing the cancer surveillance protocols in the two countries. Toward this goal, an Indo-American Cancer Consortium was conceived. The consortium brings together global transdisciplinary teams of basic cancer researchers, oncologists, epidemiologists and surveillance experts who are well equipped with the experience, diagnostic and therapeutic tools, infrastructure and collective resources. The participating institutes include Government and non-Government organizations: Manipal Academy of Higher Education, Saroj Gupta Cancer Centre and Research Institute, and Tata Memorial Centre in India, and the Mayo Clinic Cancer Center, and University of Kentucky Markey Cancer Center in the United States. The genesis of the consortium dates back to 2017. In person visits of Markey Cancer Center teams and Mayo Clinic faculty to the cancer research institutions in India, and joint cancer conferences held in 2017 and 2018 provided strong motivation to formalize the interactions by founding this Consortium. Facilitated by monthly planning meetings beginning mid-2020 and a first retreat in December 2020, held virtually due to COVID-19 travel restrictions, the consortium has been regularly organizing various interactive sessions. These include fortnightly research presentations by faculty and trainees of participating institutions, invited guest lectures from other institutions, and a quarterly mini-symposium in topical areas of cancer. Concerted efforts to mentor the next generation of scientists saw the introduction of didactic courses in basic cancer biology, translational oncology and epidemiology, for Masters, Ph.D., Pharm.D., and MD students offered by Markey Cancer Center faculty; a certificate Program in Precision Medicine: Oncology Genomics; and a virtual tumor board to guide oncologists on treatment options for precision oncology. These activities led to organic multidisciplinary collaborations involving teams of oncologists, basic scientists, pharmacists, pathologists, and epidemiologists focused on research projects. When the travel restrictions are lifted, there will be an exchange of basic science and clinical faculty and students across the participating institutions to further foster and strengthen the partnership in cancer research and training. The consortium initiatives, supported by the parent institutions and a CRDF-Global-NCI Cancer Research Training Travel Award to exchange knowledge and enable technology transfer, have already resulted in joint peer-reviewed publications. Thus, through its multi-faceted research, education, training and outreach programs, the consortium will build infrastructure, expand capacity and strengthen global cancer research leadership and healthcare at the participating institutions.

## COMMENTARY

The significance of collaborative team science cannot be overstated. Unlike the days when scientists worked in silos, and laboratory bench findings were rarely translated to the clinic, the era of team science in the last few decades have underscored the value of interdisciplinary teams of investigators with diverse expertise to tackle critical questions of clinical relevance. Such an approach is particularly essential in cancer care, where, for instance, intricate details about the genetic mutations in the patient’s tumor requires the combined expertise of basic and translation scientists as well as clinicians, to not only select the precise treatment for the patient but also maintain a readily available backup strategy in case the tumor develops therapeutic resistance.

Cancer is the second leading cause of mortality worldwide. According to GLOBOCAN, there were an estimated 19.3 million new cancer and about 10 million cancer deaths worldwide in 2020 [1]. There are currently around 1.3 million people with cancer in India, and 2.3 million in the United States [1], and the projected total cancer incidence will increase from 2020 to 2040 by almost 30% [2]. In India, the total cancer burden is projected to double to about 2 million cases by 2040 [3]. As the United States has almost double the number of cases relative to India that has a much larger overall population, it is necessary to ascertain whether these differences reflect underlying lifestyle factors and genetic effects and not simply variation in cancer surveillance protocols. Despite the heavy financial burden of cancer treatment, the net gain in patient survival is relatively short [4]. In the United States, cancer treatment is currently moving toward personalized care and some of the costs for genetic tests and treatments are covered by health insurance [5, 6]. However, in India, much more attention is required in personalized oncology with supportive insurance policies for judicious patient care. The potential to address such overarching cancer healthcare questions of societal value constitutes an obvious long-term advantage that is offered by a partnership of Indo-American institutions working together rather than the involved organizations working on their own.

Strategies to improve the impact of cancer research for patients have been widely discussed in India and United States over the past several decades. One confounding factor that restraints progress is the complexity of the disease associated with significant biological heterogeneity among cancer patients with diverse genetic predisposition, lifestyle, and environmental factors. Another impediment is the translation of basic research findings imposed by structural, financial and logistic limitations in facilitating interactions and workflow from the laboratory to the clinic. Some of these issues can be addressed by cohesive multi-disciplinary teams of basic and translational scientists and oncologists working together through multi-institutional cooperation to develop bench-to-bedside projects complemented by epidemiology data and community outreach efforts. Such teams are likely to be more effective in motivating scientific and administrative policy decisions that positively impact patient care. Based on this ideology, the Indo-American Cancer Consortium was formed by academic institutions in India and the United States. These institutions include (in alphabetical order) Manipal Academy of Higher Education (India), Markey Cancer Center, University of Kentucky (USA), Mayo Clinic Cancer Center (USA), Saroj Gupta Cancer Centre and Research Institute (India) and Tata Memorial Centre (India).

This is the first collaborative initiative involving multiple government-supported and non-government cancer centers from India and the United States. The purpose of this consortium is to develop meaningful collaborations among the participating faculty of the cancer centers in key areas of cancer research and treatment, education, and community outreach activity. The partnership is expected to bring together stakeholders with interest in basic cancer biology, translational and precision oncology, cancer prevention programs and public health policy related to cancer. The consortium has identified several thematic areas to allow physicians, scientists, health care workers, and trainees to work together toward the common agenda of cancer research, education, and healthcare. The research collaboration of the cancer centers leverages each institution’s strengths to develop premier research programs focused on key types of cancers that are most prevalent in the United States and India. Even though the interactions are primarily academic, the participating institutions have entered into a mutually acceptable confidentiality agreement to protect intellectual property. Any exchange of research materials is conducted under separate inter-institutional Material Transfer Agreements. This format enables the collaborations to jump-start without initial impediments, and a more involved Memorandum of Understanding (MoU) is developed based on the scope of each project.

Working together, the cancer centers in the consortium form an integrated interdisciplinary network. Strong inter-institutional cooperation, cutting edge research concepts and state-of-the-art research infrastructure provide an excellent environment for translational and clinical research, as well as for mentoring of MSc, PhD, PharmD, and MD trainees and junior faculty. The consortium focuses on three themes: Theme 1, Cancer Biology; Theme 2, Translational Oncology; and Theme 3, Cancer Epidemiology, Surveillance, and Community Outreach, and brings together over 30 faculty with active research programs in basic science, clinical and translational research, and public health sciences related to cancer. The consortium faculty are chosen for each theme using criteria that include the strength of their research programs, history of mentoring domestic as well as international students and postdoctoral fellows, activity in co-publishing with other program faculty, and most importantly, passion to facilitate multidisciplinary training in global cancer research.

The research aims incorporate plans to: (1) Identify critical signaling molecules, pathways, and mechanisms (Basic Sciences); (2) Discover new compounds and repurposed drugs that target key proteins involved in treatment resistance and discover biomarkers for treatment response (Translational); (3) Develop new or repurposed drugs, or combine existing drugs for clinical trials (Clinical); (4) Enhance cancer surveillance and conduct cancer disparities research to promote cancer prevention behaviors (Epidemiology, Community Outreach). These aims support the objectives of: (a) Mentoring the next generation of medical oncologists interested in academic careers in cancer to become active participants in interdisciplinary cancer research; (b) Enhancing the academic and clinical success of clinicians, scientists, and research faculty; (c) Providing life science, cancer biology or pharmacy track students the requisite didactic training in the basic science of cancer and translational science of cancer biology and epidemiology including opportunities for interaction with clinicians/oncologists; (d) Strengthening global cancer research leadership and mentorship at the participating institutions.

Prior visits of Markey Cancer Center teams and Mayo Clinic faculty to the cancer research institutions in India, and the intellectually stimulating breast cancer conferences organized jointly by the Markey Cancer Center, Manipal Academy of Higher Education and Tata Memorial Center in 2017 and 2018 provided the impetus to formalize the interactions by founding the Indo-American Cancer Consortium. The consortium activities started in mid-2020 and were facilitated by monthly virtual planning meetings. These interactions led to the first retreat in December 2020, held virtually due to COVID-19 travel restrictions, that provided an overview of the structure, organization, and programs at each of the participating cancer centers, an introduction to faculty research programs, and the consortium action plan. Since then, the consortium has been regularly organizing the following events:

i) Research presentations by faculty and students of participating institutions (once monthly). 

ii) Thematic mini-symposium (occurring quarterly). 

iii) Featured seminar series with invited guest speakers (once monthly). 

iv) Didactic courses in: (1) Basic Cancer Biology, and (2) Translational Oncology and Epidemiology, for Masters, Ph.D., Pharm.D., and MD students. 

v) Certificate Program in Precision Medicine: Oncology Genomics for faculty at participating institutions; 

vi) Virtual tumor board meetings and discussions on tumor next-generation sequencing data to guide oncologists on treatment options for Precision Oncology. 

vii) Multidisciplinary team collaborations – small group meetings involving oncologists, basic scientists, pharmacists, pathologists, and epidemiologists to develop focused research projects. 

viii) Scheduled exchange of faculty and students next year to further strengthen the partnership in cancer research and training.

The impact of the consortium is already apparent in the number of MSc, PhD, PharmD, and MD students from Manipal Academy of Higher Education, Saroj Gupta Cancer Center and Research Institute, and Tata Memorial Center registered for academic credit in cancer biology, epidemiology and therapy courses offered by the Markey Cancer Center; a CRDF-Global-NCI Cancer Research Training Travel Award to train consortium scientists from India at cancer centers in the United States; and joint research publications [7-10]. The excitement of working together across continents to promote global cancer research and training is balanced by the challenges to secure financial support for the ambitious projects of the consortium. It is envisioned that such support should be forthcoming from federal, private or philanthropic sponsors, given the value of inter-institutional collaborative programs that build infrastructure, expand capacity and enhance the research fervor and overall cancer care environment in the participating countries. Thus, the multi-dimensional programs and activities of the consortium should buttress global cancer research leadership and mentorship at the participating institutions.
